# Acute hemorrhagic encephalopathy - diagnostic challenges in a pediatric case

**DOI:** 10.1186/1471-2334-13-S1-P101

**Published:** 2013-12-16

**Authors:** Anca Cristina Drăgănescu, Monica Luminos, Magda Vasile, Anuța Bilaşco, Gheorghiță Jugulete, Angelica Vişan, Camelia Kouris, Cristina Negulescu, Cristina Popescu, Mădălina Maria Merişescu, Diana Slavu, Cornelia Dogaru, Sabina Șchiopu, Osman Endis

**Affiliations:** 1 National Institute for Infectious Diseases “Prof. Dr. Matei Balş”, Bucharest, Romania; 2 Carol Davila University of Medicine and Pharmacy, Bucharest, Romania

## Background

Acute hemorrhagic encephalomyelitis (AHEM) is considered a rare form of Acute disseminated encephalomyelitis (ADEM), due to acute cerebral vasculitis. The symptomatology consists in similar neurological findings, (meningismus, headache, seizures, multifocal neurologic signs, asymmetrical neurological deficits and coma) with rapid onset of encephalopathy and biphasic evolution. Although previous respiratory disease was registered days before this condition, establishing etiology is quite a challenge, viruses being incriminated.

## Case report

This report shows 10 months-old patient who presented with fever, seizures, multifocal neurological signs, with negative serological findings and initial favorable outcome, who developed severe neurological worsening after 3 weeks of treatment.

First magnetic resonance image (MRI) showed multifocal hyper-intense lesions affecting the CNS white matter. After 3 weeks from the initial symptoms, he had recurrence of his symptoms in association with rapidly progressive refractory status epilepticus and the second MRI showed micro-hemorrhagic lesions.

Aggressive therapeutic management was required in order to avoid the predictable fatal outcome.

